# Engaging with economic evaluation methods: insights from small and medium enterprises in the UK medical devices industry after training workshops

**DOI:** 10.1186/1478-4505-10-29

**Published:** 2012-09-03

**Authors:** Michael P Craven, Matthew J Allsop, Stephen P Morgan, Jennifer L Martin

**Affiliations:** 1 University of Nottingham, Faculty of Engineering, Tower Building, University Park, Nottingham, NG7 2RD, UK; 2 Leeds Institute of Health Sciences, University of Leeds, Charles Thackrah Building, 101 Clarendon Road, Leeds, LS2 9LJ, UK

## Abstract

**Background:**

With increased governmental interest in value assessment of technologies and where medical device manufacturers are finding it increasingly necessary to become more familiar with economic evaluation methods, the study sought to explore the levels of health economics knowledge within small and medium-sized enterprises (SMEs) and to scope strategies they employ to demonstrate the value of their products to purchasers.

**Methods:**

A short questionnaire was completed by participants attending one of five workshops on product development in the medical device sector that took place in England between 2007 and 2011. From all responses obtained, a large proportion of participants were based in SMEs (N = 43), and these responses were used for the analysis. Statistical analysis using non-parametric tests was performed on questions with approximately interval scales. Qualitative data from participant responses were analysed to reveal emerging themes.

**Results:**

The questionnaire results revealed that 60% of SME participants (mostly company directors or managers, including product or project managers) rated themselves as having low or no knowledge of health economics prior to the workshops but the rest professed at least medium knowledge. Clinical trials and cost analyses or cost-effectiveness studies were the most highly cited means by which SMEs aim to demonstrate value of products to purchasers. Purchasers were perceived to place most importance on factors of safety, expert opinion, cost-effectiveness and price. However many companies did not utilise formal decision-making tools to prioritise these factors. There was no significant dependence of the use of decision-making tools in general with respect to professed knowledge of health economics methods. SMEs did not state a preference for any particular aspect of potential value when deciding whether to develop a product. A majority of SMEs stated they would use a health economics tool. Research and development teams or marketing and sales departments would most likely use one.

**Conclusion:**

This study points to the need for further research into the education requirements of SMEs in the area of Health Technology Assessment (HTA) and also for investigation into how SMEs engage with existing HTA processes as required by assessors such as NICE.

## Background

There has been an increasing focus on economics to answer questions around efficiency and value for money in healthcare [1]. For the medical device sector, this focus has permeated through an increasing reliance on Health Technology Assessment (HTA) to ensure that the introduction of a new drug, medical device, or procedure is cost effective in line with static or shrinking health budgets in Europe and elsewhere [2]. For the medical device sector, HTA is seen as providing a means of bridging scientific evidence, the judgment of health professionals, the views of patients and the general public, and the needs of policymakers [3].

From a policy perspective, the potential value of economic evaluation to public health policy decisions, particularly at the early stages of development, includes supplementation of monitoring and assessment of innovations in horizon scanning and HTA activities, the control of technology diffusion by informing coverage and reimbursement decisions, and the direct public promotion of healthcare technologies, leading to increased efficiency [4]. In line with this subscription to HTA methodology, there has been an increase in demand for economic evidence and its evaluation from purchasers and regulators.

In the UK, one of the outcomes of the Healthcare Industries Task Force initiative [5], comprising British government, health professionals, and representatives from the medical technology industry, was to bring health economics (HE) considerations into purchasing guidance by the creation, in 2005, of the Centre for Evidence-based Purchasing (CEP) out of the more technically oriented Device Evaluation Service (DES). More recently the Medical Technologies Evaluation Programme (MTEP) of the National Institute of Health and Clinical Excellence (NICE) has taken over the remit of CEP with the aim of helping the National Health Service (NHS) in England and Wales adopt efficient and cost-effective medical devices and diagnostics more rapidly and consistently [6]. This technology evaluation programme demands that manufacturers provide their own evidence of economic value from existing literature or from de novo economic models in addition to the usual clinical evidence. The MTEP programme requires at least a cost-consequences analysis to be conducted where cost and clinical benefits are tabulated side by side so that decision makers can make judgements about costs associated with the benefits. However, cost-effectiveness is explicitly absent from NICE’s existing Interventional Procedures Programme where device-related procedures have been typically submitted for evaluation before the existence of the MTEP, so this is a considerable change for manufacturers of medical devices. Furthermore it has also meant changes for the evaluation organisations involved, several of which have provided services throughout the transition from DES to NICE.

The NHS has also been striving to improve its level of innovativeness through the efforts of the National Innovation Centre, the National Technology Adoption Centre and through programmes such as QIPP (Quality, Innovation, Productivity and Prevention). This has resulted in the widening of interest in HE amongst healthcare organisations more generally. In the USA for example, comparative effectiveness research focuses on the generation and synthesis of clinical evidence to compare the benefits and harms of alternative methods to address a clinical condition or improve the delivery of care. Its introduction and applicability to “real world” settings raises the question of the need for wider understanding of HE methods for those who may wish to articulate the cost impact of selected treatments on health systems, and for companies that are providing products associated with those treatments [7].

Whilst the demand for economic evaluation in medical device development is growing, there has also been thinking around how to integrate findings from HE work into practice. It has been acknowledged that there are outstanding problems around the incorporation of HE methods into the medical device sector which has inhibited the use of more established methods common in the evaluation of pharmaceutical products. For example, the nature of ongoing product modifications that are experienced by devices during development means that there is unlikely to be a ‘steady-state’ period where a device could be evaluated in a traditional setting such as a randomised controlled trial [8]. Further to this, a new therapy involving a device may have a wider financial implication for a healthcare provider organisation that is more difficult to forecast [9]. From the provider perspective, an interactive process in which clinical and economic estimates are used alongside emerging evidence from actual use has been highlighted as one means of addressing the issue [10]. Even so, there is ongoing discussion about the construction of such estimates (e.g. should the public or experts be consulted in the construction and elicitation of quality of life estimates) [11]. These details may not be so much of a concern if companies are able to apply “rough and ready” methods at very early stages of development [12] and if assessors are accommodating to such practices [13]. While research explores the need to strengthen modelling for devices and plans new ways to incorporate emerging evidence into analyses [8], there is still a need to establish how manufacturers and the medical device industry incorporate economic evaluations of new and existing products to articulate their value to purchasers and regulators.

In the UK, the main government agency for funding research and training in engineering and the physical sciences, the Engineering and Physical Sciences Research Council (EPSRC), is supporting multidisciplinary applied research in medical device evaluation. This involves knowledge transfer of HE methods and tools to the healthcare technologies industry. In a short, exploratory study conducted by the authors, linked to a series of workshops for the medical device industry, participants completed a questionnaire to provide insight into the self-reported levels of HE knowledge (in terms of assessing whether procedural knowledge of health economics was rated as high or low), strategies currently used by small and medium-sized enterprises (SMEs) to assess and articulate the value of new technology, and factors perceived by SMEs to be of importance to purchasers in the context of appraising technology. The insight sought from this research is current practices of SMEs and how this can be used to inform wider efforts in the education available to industry. Whilst SMEs can draw on health economics consultancy and other providers of such services, it is the in-house practices that are examined in this paper.

## Method

This research adopted an exploratory approach to investigate HE knowledge of participants attending workshops for the medical device sector on product development, and identify factors that they perceived as important to purchasers. This was achieved through the analysis of participant responses to paper-based questionnaires that were administered following a workshop. As one part of a university-led research programme in evaluation methods and tools for medical device innovation, the authors hosted a range of half-day workshop events, lasting around four hours each, between 2007 and 2011. The workshops were free to attend, and were designed to introduce medical device manufacturers to tools for use in product development. The tools included a user requirements guide to support user testing and requirements gathering [14], and a spreadsheet tool for early economic assessment of medical devices [15], which were introduced and discussed within the context of clinical case studies. Industry views about involving users during development have been reported on elsewhere [16].

In total, 12 workshops took place, provided to members of industry networks, and trade associations. The events were hosted by the Association of British Healthcare Industries trade association, and four of the regional industry network partnerships (Medilink East Midlands, Medilink West Midlands, Yorkshire & Humberside Medilink and the South East Health Technology Alliance). The marketing for all events was focused on attracting participants from SMEs and innovators that were looking to focus on uptake of their products by the NHS.

Due to resource and time constraints, data was gathered at five of the workshops, which comprises the data presented in this paper. Data was captured from workshops based in the East Midlands, Yorkshire and Humber and South East of England, although participants who attended the workshops were based in companies outside of the geographical boundaries. Data from two workshops hosted by Medilink East Midlands were included in this paper. The questionnaires were provided to the participants at the end of the workshop session, so that responses relating to HE knowledge prior to the workshop could be informed following discussion of the topic. The questionnaires were administered by a researcher not involved in the delivery of the content for the workshop. The questionnaires outlined that information provided was to be used for academic research into the needs of industry and adopters of innovations within the NHS, is confidential, and that reported findings from the research will not refer to named organisations.

The questionnaire contained six sections: i) description of participants’ organisation and their role within it; ii) self-rating of their procedural knowledge of HE prior to the workshop (using 5-point scale from ‘none’ to ‘expert); iii) participant identification of any formal decision-making tools that are used within their organisation to support product development (e.g., strategic and financial valuation of projects, weighting and scoring of products and product criteria) iv) participant rating of the importance of six decision-making priorities when initiating medical device development, using a 5-point scale from ‘very important’ to ‘not important’ (anticipated profit margin, market competition, enthusiasm of customer for a device, purchasers opinion, expert opinion, uniqueness of the technology); v) participant rating of how important they thought each of seven factors were to purchasers when assessing a product during procurement, using a 5-point scale from ‘very important’ to ‘not important’ (device price, cost effectiveness, expert opinion, patient group opinion, safety of the product, company reputation, environmental impact); vi) participants description of how they would aim to demonstrate the value of medical products to the NHS or other healthcare purchasers (e.g., clinical trial, key opinion leaders, cost-effectiveness calculation); vii) whether participants envisaged that a spreadsheet tool for early economic assessment of medical devices could be utilised by their company (responding with the options ‘yes’, ‘maybe’, or ‘no’), and were asked to list who would be likely to use it. The factors included in questions around decision-making priorities and purchaser decisions during procurement were generated through discussions within the research team, drawing on expertise in the development and purchasing of medical devices. Qualitative data from participant responses were analysed to reveal emerging themes, presented as frequencies or indicative text. Where appropriate, responses were compared against five levels of HE knowledge as the independent variable. Statistical analysis using non-parametric tests was performed on questions with approximately interval scales. Where statistical analysis was required for responses, IBM® SPSS® software was used.

## Results

### Organisations and role of participants

Of the total number of participants who attended the five workshops (N = 69), 62% were from SMEs (N = 43) (see Table 1). The remaining participants were from larger companies, universities or health providers. Although all participants from SMEs completed a post-event questionnaire, responses were not provided by participants for all of the questions. The majority of participants from SMEs held the role of managing director or director in the company in which they were based, with the role of director covering responsibilities across a range of activities, including marketing, new product development, and commercial roles.

**Table 1:** Breakdown of the job roles of workshop participants attending the workshops

**Job role**	**Number (%)**
Director	17 (39.5%)
Managing Director	16 (37.2%)
Manager	2 (4.7%)
Product Manager	2 (4.7%)
Senior Project Manager	1 (2.3%)
Company Secretary	1 (2.3%)
Senior Partner	1 (2.3%)
Consultant	1 (2.3%)
Not Defined	2 (4.7%)

### Health economics knowledge

**Figure 1:**
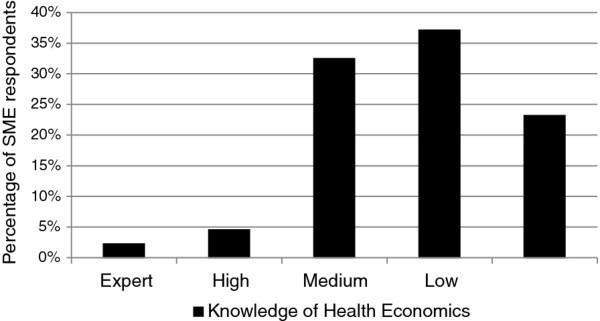
Knowledge of health economics amongst workshop participants (N = 43).

Of the participants from SMEs, 23% of respondents reported having no, or almost no, knowledge of HE, 37% reported a response of *low* HE knowledge, 33% *medium*, 5% *high* and 2% *expert* (Figure 1).

### Use of formal decision-making tools within participant companies

When asked to identify any formal decision-making tools that are used within their organisation, 42% of respondents (N = 40) said that they were using at least one form of tool in their business. Amongst the respondents, stage-gate methods, technology roadmaps and HE were mentioned as formal methods. Other responses indicated the use of formal methods without specifying them. Amid the reported HE methods used, Monte Carlo sensitivity simulations (a financial planning approach that analyses multiple projections using a range of possible return rates), basic HE models and use of Quality-adjusted Life Years (QALY) (a metric that takes into account both the quantity and quality of life generated by healthcare interventions and is the arithmetic product of life expectancy and a measure of the quality of the remaining life-years [17]) were highlighted. Quantitative market research methods were also described as formal by some respondents. Decision methods described as informal by participants included prior experience, boardroom decisions and decisions made on criteria of market analysis/user demand and practical means of delivery, also including assessment of intellectual property protection.

Table 2 shows that the use of formal decision-making tools was greater for those participants reporting *medium*, *high*, and *expert* HE knowledge, with greater proportions of those reporting *low* to *none* HE knowledge also being non-users of formal decision-making tools. The relationship between these variables was assessed, but no significant relationship was found (τ = −0.138, p ≥ 0.05).

**Table 2:** Knowledge of Health Economics vs. Use of Formal Decision-Making Tools

**Knowledge of Health Economic**	**Use of Formal Decision Making Tools (N = 40)**	
	**Yes**	**No**
None	4 (44%)	5 (56%)
Low	4 (27%)	11 (73%)
Medium	7 (54%)	6 (46%)
High	1 (50%)	1 (50%)
Expert	1 (100%)	0 (0%)

### Decision-making priorities

When probing companies’ motivating factors for developing a new product using six factors (*anticipated profit margin, market competition, enthusiasm of the customer for the device, purchaser opinion, expert opinion and uniqueness and readiness of the product*) a Friedman test was used to analyse variance in the participant responses (N = 32) by ranks. However, the analysis revealed no significant differences in the ranking of these six factors (*χ*^2^ (5) = 7.00, p ≥ 0.05).

### Participant rating of factors perceived to be of importance to purchasers

To examine the participants’ ratings of factors perceived to be of importance to purchasers when assessing a product during procurement (N = 39), responses were ranked using the Friedman test which highlighted an overall statistically significant difference between the mean ranks of the related groups (*χ*^2^ (6) = 88.26, p < 0.05). Table 3 displays the average ranks of the different categories according to their perceived importance to purchasers.

**Table 3:** Factors of importance to purchasers, as rated by the workshop participants

**Category**	**Rank**
Safety of the Product	2.89
Expert Opinion	2.97
Cost Effectiveness	3.12
Device Price	3.32
Company/Brand Reputation	4.51
Patient Group Opinion	5.32
Environmental Impact of a Product	5.85

From these tests we were able to rank the six factors. *Safety of the product, Expert opinion*, *Cost-effectiveness* and *Device price* were found to be similarly ranked (mean rank between 2.89 and 3.32) with no statistical difference between these factors. This was followed by *Company/brand reputation* and *Patient group opinion* which were both ranked significantly lower than all of the top four factors, but not significantly different from each other (ranking 4.51 and 5.32 respectively). Lastly, *Environmental impact* had the lowest rank that was significantly lower than all other factors (mean rank 5.85).

### Participants’ description of how they aim to demonstrate the value of medical products to the NHS or other healthcare purchasers

Workshop participants were asked to put in words how they would demonstrate value to purchasers. Table 4 documents explicit mention of methods used by the participants (N = 43), which were accompanied by more general references to evidence-based medicine, cost-benefit analysis to healthcare providers and patients, and statement of benefits. The responses documented in Table 4 were coded from qualitative responses, with a varied number of responses provided by individual participants.

**Table 4:** Methods currently used by delegates to demonstrate value to purchasers

**Methods used for demonstrating value**	**Number of delegates**
Clinical trials (external and internal)	11
Cost analysis and cost-effectiveness studies	10
Outcomes Survey	4
Demonstrate public and patient benefit	4
Comparative studies	2
Demonstrate benefits to user	2
Opinion leader support	2
All factors listed in the questionnaire	2
Demonstrate product quality	1
Demonstrate benefit to healthcare provider	1
Peer-review publications	1
Seminars / demonstrations	1

To provide more insight into the descriptions provided by participants about how their company aims to demonstrate the value of medical products to purchasers, Table 5 outlines the exact responses of a selection of participants.

**Table 5:** Participant responses about how their company aims to demonstrate the value of medical products to purchasers

**Statement**	**Role in company**	**HE knowledge**
“Comparative studies by experts”	Director	Low
“…through products effectiveness, values, and how it can help improve users and patients life”	Product Manager	Low
“Show the data on public health impact”	Consultant	Low
“Seminars, demos”	Director	Medium
“By demonstrating product quality”	Senior Partner	Medium
“Business model based on PSSRU figures, clinical benefits, peers review formal publications”	Director	High

It is apparent from reviewing the comments that there is diversity in the approaches applied by SMEs and no clear consensus was evident in the reporting of methods used to demonstrate the value of their medical products is present.

The last question on the questionnaire asked participants whether they would consider using a HE spreadsheet tool in their company and who would be likely to use it. From the responses gathered (N = 39), 67% of participants indicated that they would be likely to use such a tool, 26% stated maybe, and 7% said that they would not use such a tool. For those participants who identified that they would be likely to use such a tool, it was highlighted that there would be scope to apply the tool in research and development teams, marketing and sales departments, or both within their companies.

## Discussion

Amongst participants from SMEs attending training workshops tools for use in product development, HE was perceived as of importance to purchasers, and is entering into decision-making processes by SMEs in the medical devices industry. Prior knowledge of HE was such that 2 in 5 of the participants already had at least medium knowledge. Cost-effectiveness was ranked by SMEs to be amongst the top factors influencing purchasers, and 92% of respondents said that their company would use or might use a HE tool. At the same time, less than half of the companies reported using formal methods of decision support during product development. In terms of demonstrating the value of a product, clinical trials appear to dominate as the main way of doing this, alongside cost-effectiveness which was mentioned by almost 1 in 6 participants in those applying formal methods.

The findings of this paper suggest that, for those participants from SMEs who attended the workshops, there is an awareness of the increasing use of HE by purchasers in decision making. However, the varying levels of HE knowledge reported by participants from SMEs, and the lack of HE in decision making during product development suggests that attention may need to be given to education needs, and tools to support the application of HE. There is currently no research that examines the most effective means of educating those from industry about HE and the wider coverage of HTA, so this is also an area for future research.

Taking a global perspective in their report on HTA of medical devices, the World Health Organisation [18] outline that disseminating knowledge and skills in HTA should be based on a progressive strategy. The suggested first stage is the identification of individuals with a capacity for accessing and understanding HTAs, and then making these individuals the “focal points” of HTA and scientific evidence of effectiveness of healthcare interventions. The “focal points” could be located in a national research organisation, government, a university or other non-profit agencies concerned with advancing the use of HTA for good governance in policy and decision making. Their role would be to facilitate and mobilise knowledge around HTA, using such methods as organising or facilitating meetings, providing presentations and conducting workshops. The WHO report draws on research from Romania [19] in which it is pointed out that success in implementing a HTA presence in a country setting depends on factors such as local political, economic, and educational support. The multidisciplinary and interdisciplinary nature of HTA sees HE amidst its array of components and, as such, how it is taught to and used by manufacturers feeds into the wider implementation of HTA. With well-established HTA bodies in the UK, it is a useful testing ground to understand how best manufacturers and developers of medical technology can adopt and implement HE within an environment in which there are increasing calls for its use. The progressive strategy outlined by the WHO may provide an opportunity to assess the agenda for increasing knowledge around HE within the context of HTA, alongside providing understanding on how best to disseminate knowledge to support HTA development more widely.

In the UK, processes such as the NICE Medical Technologies Evaluation Programme do now require the formalised use of economic evaluation methods such that submissions by industry are expected to perform at least a cost-consequences analysis. Although process and methods guides are provided for those submitting technologies to this programme, the indication in this research that some companies would use a spreadsheet tool for early economic evaluation, in particular those who see scope for its use in research and development, indicates that there may be scope for policy makers to engage with industry through the generation of spreadsheet tools, such as those that are generated by NICE as costing templates as part of implementation support.

Whilst this research has generated an initial insight into the knowledge and awareness of HE use within HTA for the medical device sector, it is acknowledged that the sample size was inadequate to extrapolate out to represent SMEs throughout the whole UK medical device sector. Further research is required to identify the extent of HE knowledge within SMEs and to develop methods to support the education and implementation of HE within the HTA landscape for medical devices in the UK. As a starting point, research has shown that those developing innovative medical devices struggle to express the value of their work because they only engage with HE at the later stages of development [20].

Any strategy around improving knowledge of HE within the medical device industry should act on research showing the benefits of its inclusion at the early stages of development [21]. Although there is little research on the education of the medical device industry, an approach to HE teaching in practice could benefit from developing collaborations between basic and clinical researchers from academic institutions on the one hand, with engineers and scientists from the research divisions of device and pharmaceutical companies on the other. Such links have been encouraged by the European Society of Cardiology [22] as a way of devising legitimate and ethical collaborations between healthcare providers, academic institutions, professional associations, charitable foundations, and industry to support continuing medical education and facilitate some of the best and most innovative research ideas. However, indicative of the ever-increasing range of stakeholders involved in health research, there is need for closer examination of diverse groups involved in this process to understand the best way to meet their various needs.

This study points to the need for sustained effort in education of HE so that its principles, and the benefits it can deliver, are at the finger-tips of industry when they are preparing their evidence. This would allow continued impact of cost-effectiveness in its position of supporting healthcare decision makers, particularly in terms of its role for informing technical issues around clinical policies [23]. Whilst there have been guidelines developed for performing health economic evaluations [24,25], their contribution to decision-making is through definition of a minimum methodology to be applied when forming or reviewing an economic evaluation. Although this provides support to align expectations of SMEs and healthcare providers regarding the construction of economic evaluations, the process of deciding whether to adopt an innovation is not prescribed. A similar issue extends out more widely into HTA, where the structures, processes and mechanisms by which technology coverage decisions can and should be made in healthcare have also been highlighted as requiring further research [26]. For HE, there is scope to explore providers’ decision making, and particularly to gauge the importance of economic evaluation within this, which could in turn impact on the uptake, construction, and presentation of health economic evaluations by SMEs.

The participants discussed in this paper had attended a workshop hosted by those conducting the research which aimed to provide information on both health economics and user needs. Whilst it is acknowledged that the sampling strategy may have impacted the research through a selection bias, the researchers emphasised confidentiality to participants and, given the scarcity of published industry views in this area, were keen to use the collection of workshops as a means of interacting with industry for this research. Additionally, an interest in HE may have been a cause for participants to register for the workshop, and the training provided in HE during the workshop may have helped participants realise its importance. Future research could consider alternative approaches to access SMEs, either through targeted interviews or a broad survey of trade association memberships. There is scope for more research in the area to document the extent of need for HE education in industry, through both increasing the sample sizes involved, and by generating comparisons with other company types within the medical device sector.

## Conclusion

This study points to the need for further research into the education requirements of SMEs in the area of Health Technology Assessment and also for investigation into how SMEs engage with existing HTA processes as required by assessors such as NICE. Furthermore, tools to support economic evaluation are of interest to the medical devices industry. It is hoped that the findings presented will generate support in bridging the gap between increasing demands for health economics evidence by policy makers and the delivery of responsive information and self-evaluation of cost-effectiveness of products by SMEs in the medical device sector.

## Competing interests

The author(s) declare that they have no competing interests.

## Authors' contributions

MC and JM were involved in the design of the questionnaire and data collection. MC and MA performed statistical analysis of the data and prepared the first and subsequent drafts. SM was involved in refining the drafts and guiding the study development. All authors read and approved the final manuscript.
